# Lymphoma Involving the Heart: A Case Report

**DOI:** 10.3389/fcvm.2020.00027

**Published:** 2020-03-17

**Authors:** Randa Tabbah, Elissar Nohra, Rachoin Rachoin, Kabalan Saroufim, Bassam Harb

**Affiliations:** ^1^Department of Cardiology, Holy Spirit University of Kaslik, Jounieh, Lebanon; ^2^Department of Internal Medicine, Holy Spirit University of Kaslik, Notre Dame de Secours, Jbeil, Lebanon; ^3^Head of Department of Echocardiography at Notre Dame de Secours, Jbeil, Lebanon; ^4^Pulmonary and Critical Care Medicine, Holy Spirit University of Kaslik, Notre Dame de Secours, Jbeil, Lebanon; ^5^Interventional Cardiologist, Notre Dame de Secours, Jbeil, Lebanon

**Keywords:** primary, lymphoma, pleuropericarditis, right heart, cardiac tumors, case report

## Abstract

**Background:** We describe a rare case of cardiac malignancy that counts for <1% of the primary cardiac tumors with a poor prognosis.

**Case summary:** It's a rare case of a primary cardiac lymphoma in a 59-year-old patient who presented for recurrent pleuropericarditis with low-grade fever and night sweats. Investigations revealed an infiltrating mass in the right atrioventricular groove involving the right atrium and the right ventricular free wall. Pathology confirmed the diagnosis of non-Hodgkin's lymphoma. Chemotherapy with R-CHOP regimen was a success, but the patient suffered from recurrence with a complete remission after a second chemotherapy cycle. He was a candidate for bone marrow transplant to reduce other possible relapses.

**Discussion:** Early diagnosis is better for long-term prognosis and improves quality of life.

## Introduction

Cardiac tumors are most commonly benign, counting for 75% of the primary cardiac masses. These masses are potentially lethal whether they are malignant or benign. Lymphomas and sarcomas are the most common malignant ones (1). They affect elderly men with a late and poor prognosis (2). One of the rarest heart tumors is non-Hodgkin's lymphoma. The most common type is the diffuse large B cell lymphoma. This type of tumor needs to be treated on-time, due to its fatal prognosis. Early diagnosis is mandatory (3–5).

## Case Presentation

A 59-year-old male presented with a 3-month history of flu-like illness, palpitations, and atypical chest pain irradiating to the shoulders. The patient also noted also a decrease in his appetite with progressive weight loss of 4 kilos in 1 month.

1 month before admission, he had an exacerbation of his symptoms, with a new onset of cough, dyspnea increasing with inspiration, and chest pain improving when bending forward. In addition, he had some rare episodes of low-grade fever with night sweats.

The patient's past medical history revealed asthma, chronic sinusitis, and acute intoxication with paracetamol. When he first consulted his physician, he was diagnosed with pericarditis and a right pleural effusion treated with colchicine (1 mg, 1 tablet daily), and steroids (0.25 mg/lg, then 0.5 mg/kg).

An EKG showed a slow atrial fibrillation of 50 beats per minute, with low voltage QRS. A chest X-ray revealed a bilateral pleural effusion with cardio mediastinal enlargement.

A CT scan showed a moderate bilateral pleural effusion, with a pericardial thickening of 5 mm and mild pericardial effusion.

A pseudo-intrapericardial mass in the right anterior mediastinum and centered on the right coronary artery with multiple mediastinal adenopathy was also described.

This presentation encouraged the physician to search for granulomatous lesions or tuberculosis or even to think about Wegener's disease, but malignancy was also an option.

An additional abdominal ultrasound was suggestive of hepatomegaly.

An ultrasound-guided thoracentesis of the pleural effusion was done, suggesting an exudative effusion indicating malignancy, especially lymphoma.

Transthoracic echocardiography showed an effusive-constrictive pericarditis pattern. A decrease of >25% in the peak E wave velocity by pulsed wave doppler, due to a respiratory variation of the mitral inflow velocity was noticed, in addition to a visually respiratory phasic shift of the interventricular septum toward the left ventricle cavity during inspiration with an early diastolic mitral septal annulus velocity. Furthermore, an expiratory diastolic flow reversal of the hepatic vein is seen with a mild to moderate circumferential pericardial effusion. These findings encouraged the physician to perform more investigations with a transesophageal echocardiography.

A transesophageal echocardiography revealed a mild circumferential effusion with a thick pericardium. An infiltrating mass in the right atrioventricular groove involved the right atrium and the right ventricular free wall, extending to the posterior free wall of the right atrium and probably the interatrial septum. The superior vena cava appeared thick also and probably infiltrated, as for the right pulmonary artery.

**Figure 1:**
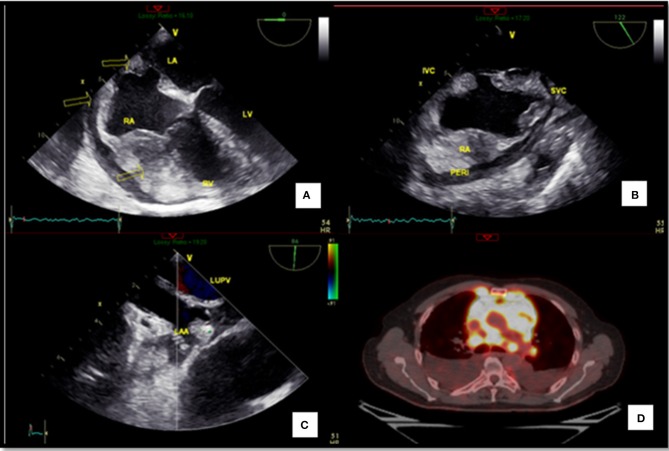
Extension of the lymphoma**. (A)** Transesophageal echocardiography revealing right ventricular infiltration. **(B)** Transesophageal echocardiography bicaval view showing an infiltration of the right atrium. **(C)** Transesophageal echocardiography (left atrial appendage with a dense round image that could be a thrombus). **(D)** PET-CT revealing the extension of the lymphoma. RA, Right atrium; RV, Right ventricle; LA, Left atrium; LUPV, Left upper pulmonary veins; LAA, Left atrial appendage; IVC, Inferior vena cava; SVC, Superior vena cava; PERI, Pericardium.

A decrease in the emptying velocity <25 cm/s of the left atrial appendage with a suspicious thrombus was noticed. A pattern in favor of a primary cardiac tumor was most likely (Figures 1A–C).

MRI may be used for evaluation of cardiac masses, extracardiac structures, involvement, and characterization of masses in the differentiation of tumors from intracavitary mural thrombi. It also helps in discriminating between benign versus malignant cardiac masses, emphasizing infiltration, invasion, and other signs of metastasis; but in our case, we opted for PET scan, being the gold standard in this area. Primary cardiac lymphoma can be hypointense on T1-weighted imaging and hyperintense on T2-weighted imaging, but it can demonstrate heterogeneous signal intensity. Contrast enhancement is frequent and may be homogenous or heterogeneous.

A PET scan was done to study the extension of the tumor. A high-fatty diet with low carbohydrates 24 h before PET (no cereals, pasta, dry beans, fruits, sugar, honey, candy, desserts, starchy vegetables, alcohol, bread, rice, gravies, jams, milk, or caffeine) was initiated. The patient fasted for 6 h. There was no need to suppress the physiological myocardial uptake.

The patient was positioned ~60 min after the intravenous administration of 8.3 mCi of FDG (fluorodeoxyglucose uptake). A non-contrast CT scan was acquired from the skull base to the mid thighs. A 3D emission scan of the same area was acquired. The patient's fasting glucose level was 94 mg/dl.

The PET scan showed a cardiac mass infiltrating the right heart cavities and the interatrial septum, involving the pericardium. Diffuse metastatic mediastinal adenopathy with bilateral pleural effusion and metastatic pleural implants was observed.

Two nodules in the upper lobes with bilateral cervical adenopathy and a right supraclavicular adenopathy and a retroperitoneal adenopathy with omental thickening and peritoneal implants in the pelvis were observed. It also revealed bilateral adrenal lesions with right renal mass. These findings were suggestive of cardiac sarcoma or cardiac lymphoma of the heart (Figure 1D) (Supplementary Video 1).

**Figure 2:**
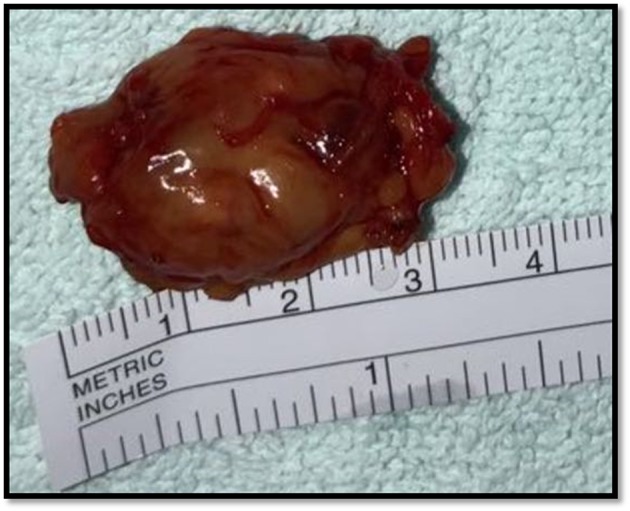
Right supraclavicular adenopathy. A specimen of the right supraclavicular adenopathy by percutaneous biopsy.

For a definitive diagnosis, a percutaneous biopsy of the right supraclavicular adenopathy was performed (Figure 2). Pathology confirmed the diagnosis of a non-Hodgkin's cardiac lymphoma.

The patient was treated with high doses of dexamethasone to relieve symptoms. He developed a severe symptomatic bradycardia and long pauses suddenly. A single chamber pacemaker VVI was implanted as a backup because the patient was in atrial fibrillation and no need for atrial lead in this case.

Even with symptomatic treatments, he complained of severe shortness of breath, due to his severe bilateral pleural effusion. Bilateral pleural chest drainage was done as a salvage procedure because the patient was in severe respiratory distress. After the procedure, he experienced a rare complication: a re-expansion pulmonary edema (6–8). He was moved to the intensive care unit (ICU) and supportive therapy was given for stabilization and symptoms relief. After recovery, he complained of a new onset of diplopia. Brain CT was done to rule out cerebral metastasis with a lumbar puncture. These exams didn't reveal any abnormalities. Eight cycles of chemotherapy with the R-CHOP protocol. Rituximab, Cyclophosphamide, doxorubicin, vincristine, and prednisolone were initiated. After these cures, a new PET CT was performed.

**Figure 3:**
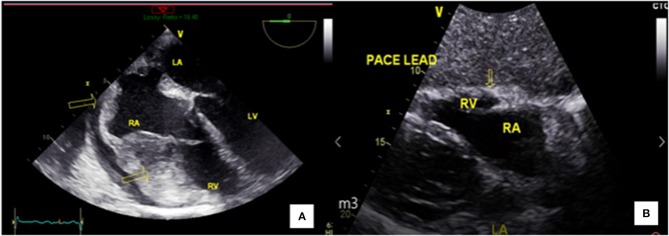
Complete remission after chemotherapy. **(A)** Transesophageal echocardiography revealing right ventricular infiltration before chemotherapy. **(B)** Transthoracic echocardiography revealing remission after chemotherapy.

Complete metabolic treatment response was noted with a persistent focal prostatic uptake at the right base. An echocardiography declared a complete remission. There were no more infiltrated tissues, and the right ventricle regained its function (Figure 3).

Few months later, the patient came to the hospital complaining of an external chest pain with palpation and no dyspnea or any other symptoms. On physical examination, a palpable subcutaneous thoracic mass was noticed. A total body CT scan revealed some new large left supra- and retro-clavicular, mediastinal, and pleuropericardic masses. The largest one was 6 cm. A PET scan confirmed a recurrence of his lymphoma. Three large hypermetabolic mediastinal masses with cervical, mediastinal, axillar, and hilar lymphadenopathy were observed. An invasion of the subpleura with a pulmonary parenchymal focus was also noticed.

Two subcutaneous nodules were detected in front of the right pectoral muscle with a left pleural effusion and a markedly increased uptake at any site including new site of disease (Deauville score 5). A new cycle of chemotherapy began.

Two months later, a PET scan for control was done with complete remission. Two weeks after, the patient was sent for bone marrow transplant to prevent further recurrences. The patient remained actually stable.

## Discussion

This patient presents a rare cardiac malignancy that counts for <1% of the primary cardiac tumors. It is described as a typical non-Hodgkin's lymphoma invading the right atrium and the right ventricle to the pericardium. The diagnosis was suspected when the patient presented first with refractory pericardial effusion as in this case with the B symptoms (fever, night sweats, and weight loss) (9).

Many complications regarding this tumor are life-threatening: arrhythmias, pericardial effusions, and ventricular septal rupture. Theses complications, if left untreated, are rapidly fatal, leading to tamponade, cardiogenic shock, and death, due to refractory heart failure.

Time is muscle in these scenarios, and preventing the extension to cardiac tissues is the key for a better outcome.

In this case, the patient had several complications that were treated on time. First, atrial fibrillation was also a sign of involvement of the conduction system. Patients with arrhythmias in these cases have a median of survival of 1 month, whereas for those free from arrythmias, survival is about 6 months. In addition, pericardial effusion was present, but fortunately didn't lead to tamponade. Furthermore, this patient had many rare complications as the re-expansion pulmonary edema, an iatrogenic complication after pulmonary expansion when performing a thoracentesis or a pleural chest drainage. The mortality of this complication is 20% and the incidence count for 1% (7, 8).

In these types of tumors, palliative surgeries were needed to correct hemodynamics, as the Fontan procedure is an anastomosis of the superior vena cava to the right pulmonary artery to bypass the right heart (9). But this patient was stabilized with symptomatic and device therapy. Chemotherapy was the gold standard of care with R-CHOP protocol.

The addition of rituximab to the CHOP regimen increases the complete-response rate and prolonged event-free and overall survival in elderly patients with diffuse large B-cell lymphoma, without a clinically significant increase in toxicity which was obvious in our patient (10).

He was first diagnosed with pericarditis and treated with NSAID, but recurrent chest pain pushed the physician to further investigations revealing atrial fibrillation with electrical system invasion. A poor prognosis was predictable in this case, due to the involvement of the right heart and signs of acute heart failure. Diagnosis was made with a multimodality imaging. Transthoracic ultrasound revealed an effusive constrictive pattern. Further investigation with a transesophageal echocardiography showed a massive invasion of the right heart.

MRI could have played an interesting role in this setting, but a PET scan was a gold-standard approach and was conclusive about the disease extension. Pathology made it all clear in the end. It's a complicated case involving all cardiology fields. Early diagnosis is the key to fight this devastating illness (11–14).

## Conclusion

This is a rare case of a primary cardiac lymphoma of the heart that survived many complications from a recurrent pleuropericarditis and damage of the conduction system of the heart, followed by a re-expansion pulmonary edema. A complete remission, two times after chemotherapy, was noticed. A multimodality imaging was needed in this setting to clarify the diagnosis for an earlier and better outcome. Earlier diagnosis is better for long term prognosis and better quality of life.

## Data Availability Statement

The raw data supporting the conclusions of this article will be made available by the authors, without undue reservation, to any qualified researcher.

## Ethics Statement

Written informed consent was obtained from the individual(s) for the publication of any potentially identifiable images or data included in this article. Patient consent form was read and signed by the patient.

## Author Contributions

All authors contributed in this patient care, diagnosis and treatment, and in writing this article.

### Conflict of Interest

The authors declare that the research was conducted in the absence of any commercial or financial relationships that could be construed as a potential conflict of interest.

## Supplementary Material

Supplementary Video 1 PET-CT revealing the extension of the lymphoma.
